# Elevated Markers of Intestinal Barrier Dysfunction and TLR2 Ligands in Individuals with Type 1 Diabetes

**DOI:** 10.2147/DMSO.S612334

**Published:** 2026-09-03

**Authors:** Nafeesah Noorwali, Raphaela Staltner, Aleksejs Fedulovs, Jana Schneider, Polina Zalizko, Andris Romašovs, Ina Bergheim, Jeļizaveta Sokolovska

**Affiliations:** 1 Department of Nutritional Sciences, Molecular Nutritional Science, University of Vienna, Vienna, Austria; 2 Department of Clinical Nutrition, Faculty of Applied Medical Sciences, Umm Al-Qura University, Makkah, Saudi Arabia; 3 Clinical Research Center, Department of Clinical and Personalized Medicine, Faculty of Medicine and Life Sciences, University of Latvia, Riga, Latvia; 4 Centre of Gastroenterology, Hepatology and Nutrition, Pauls Stradiņš Clinical University Hospital, Riga, Latvia

**Keywords:** I-FABP, sCD14, TLR2, intestinal permeability

## Abstract

Studies suggest that alterations of intestinal barrier function and a subsequent increased translocation of bacterial endotoxins may be critical in type 1 diabetes mellitus (T1DM) and may contribute to the proinflammatory state of patients. If Gram-positive derived bacterial toxins are also altered in the setting of T1DM is not yet clarified. In the present study, using a SEAP reporter assay and ELISA, markers of intestinal permeability, concentration of Toll like receptor (TLR) 2 ligands and soluble CD14 (sCD14) were assessed in serum of individuals with T1DM (n=19, m: 10/ f: 9) and age-matched healthy controls (n=18, m: 3/ f: 15) from the LatDiane study. BMI and HbA1c were significantly higher in individuals with T1DM. Serum concentrations of intestinal fatty acid binding protein (I-FABP) were significantly higher in individuals with T1DM than in controls as were concentrations of TLR2 ligands and sCD14 protein. A multivariable linear regression analysis revealed an independent association of T1DM with higher TLR2 ligand levels. Our findings indicate that not only bacterial endotoxin but also TLR2 ligands are elevated in patients with T1DM.

## Plain Language Summary

T1DM is associated with chronic inflammation, but the underlying mechanisms remain unclear. We investigated markers of intestinal barrier and bacterial toxin signalling in adults with T1DM and healthy controls. In individuals with T1DM markers of intestinal barrier function and bacterial toxins eg, sCD14 and TLR2 were higher than in controls. After adjustment for age, BMI, sex, and smoking, differences were still found for TLR2 ligands.

## Introduction

Globally the prevalence of type 1 diabetes mellitus (T1DM) has remained relatively stable throughout the last decades, affecting ~10.5% of the general population world-wide in 2021.^1^ T1DM is an autoimmune disease causing the loss of ~90–100% of pancreatic β-cell function, requiring lifelong insulin therapy.^2^ Besides impaired glucose metabolism, T1DM is characterized by proinflammatory alterations such as elevated C-reactive protein, MCP-1 and IL-6^3^ and increased monocyte activity.^4^ Animal models of T1DM have shown that the development of T1DM is linked to increased intestinal permeability.^5^ Also, a meta-analysis of 14 studies found that fasting lipopolysaccharide (LPS) levels are about 235% higher in T1DM individuals compared to healthy controls,^6^ suggesting that bacterial endotoxins may contribute to the chronic low-grade inflammation observed in T1DM.^7^ Somewhat supporting this hypothesis Mohammad et al^8^ reported increased T*LR2* and T*LR4* mRNA expressions in bone marrow-derived macrophages from non-obese type 1 diabetic mice. However, whether other TLR ligands including those derived from Gram-positive bacteria, eg, lipoteichoic acid and peptidoglycan are altered in individuals with T1DM and if these are related to the pro-inflammatory state found in T1DM has not yet been clarified. Starting from this background, the aim of the present study was to examine whether markers of intestinal barrier function and circulating TLR2 ligands concentrations as detected by a SEAP reporter gene assay are altered in individuals with T1DM compared to age-matched controls.

## Methods

### Participants

All participants were part of the longitudinal LatDiane Study, initiated in 2013 at the University of Latvia as part of the international InterDiane consortium (see Ref^9^ for more details), enrolling adults diagnosed with T1DM before age 40 with insulin treatment initiated within one year of diagnosis and C-peptide levels <0.3 nmol/L^9^ as confirmed by health care professionals and multiple daily insulin injection therapy. Recruitment, biobanking and sample storage were carried out in agreement with the procedures of the Genome Database of the Latvian Population.^10^ The study was approved by the Latvian Central Ethics Committee Nr. 01–29.1/3 (dated, 10 July 2013), Nr. A-17/19-10-17 (dated, 17 October 2019), and Nr. 01–29.1/2226 (dated, 30 April 2020) and carried out in accordance with the ethical standards laid down in the Declaration of Helsinki 1964. Written informed consent was obtained from all participants of the study. Sample size calculation was performed using GPower 3.1.9.4 (GPower, Version 3.1.9.2, Düsseldorf Germany). Based on unpublished preliminary date, the estimated effect size was 0.855. Assuming an independent *t*-test, α = 0.05 and a power of 80%, a total sample size of 36 participants (n= 18 per group) was required. The final study population comprised 19 individuals with T1DM and 18 healthy age-matched controls, who were selected from the LatDiane study. Only individuals aged 21–44 years were included with diabetes duration >5 years to minimize age-related confounding effects on intestinal permeability. General exclusion criteria included chronic liver disease including steatosis as assessed by magnetic resonance imaging (MRI), use of steatogenic medications, pregnancy, inflammatory bowel disease, coeliac disease, acute inflammation, or fever. Some individuals with T1DM had comorbidities like diabetic nephropathy, retinopathy and hypertension. Alcohol consumption classified as high-risk (>10 drinks/week or >2 drinks/day for females, and >15 drinks/week or >3 drinks/day for males) was an exclusion criteria for both groups. Inclusion criteria applied for individuals with T1DM were: no use of other glucose-lowering medications. Inclusion criteria for controls were: normal renal, hepatic, and cardiac function, no hypertension, and no family history of diabetes or other chronic diseases, diabetes type 2 or prediabetes.^9^ This study was reported according to the STROBE guidelines.^11^

### Clinical Measures

In fasting serum samples, alanine aminotransferase (ALT), aspartate aminotransferase (AST), total cholesterol, low-density lipoprotein (LDL) and high-density lipoprotein (HDL) cholesterol, triglycerides, and haemoglobin A1c (HBA1c%) were assessed in a clinical routine laboratory.

### TLR2 Ligands and Enzyme-Linked Immunosorbent Assay (ELISA)

Serum concentration of TLR2 ligands were measured employing a commercially available HEK293 cell-based assay (Invivogen, Toulouse, France) as described previously.^12^ Serum protein levels of soluble cluster of differentiation 14 (sCD14) and intestinal fatty acid binding protein (I-FABP) were assessed using ELISA kits (R&D Systems, Catalog # DY383 and Catalog # DY3078; Bio-Techne Corp., Minneapolis, MN, USA) following the manufacturer’s instructions and as detailed previously.^13^

### Statistical Analysis

All measurements are presented as means ± standard error of means (SEM). Data normality was assessed using the D’Agostino–Pearson test. Differences between groups were analysed using an unpaired *t*-test. Multivariable linear regression of I-FABP, sCD14 and TLR2 ligands adjusted for BMI, sex, age and smoking was performed using R (version 4.4.2; R Core Team, 2024) with RStudio (version 2024.12.0; Posit Team, 2024) as the integrated development environment. For correlation analysis, Spearman’s correlation test was used. Analyses were performed in GraphPad Prism v7 (San Diego, CA, USA), with significance set at p < 0.05.

## Results

### Characteristics of Controls and Individuals with T1DM

Neither age nor sex distribution differed between the two groups (see Table 1). Among individuals with T1DM, 4 were normal weight and 15 were overweight, while all controls were normal weight. Body mass index (BMI), waist circumference (WC), hip circumference, waist-to-hip ratio (WHR), systolic blood pressure (SBP) and diastolic blood pressure (DBP) were significantly higher in the T1DM. None of the participants showed signs of metabolic dysfunction-associated steatotic liver disease (MASLD). HbA1c%, total cholesterol and LDL concentrations were significantly higher in the serum of individuals with T1DM than in controls, whereas serum HDL, triglycerides, ALT and AST concentrations were comparable between groups (Table 1).

**Table 1:** Characteristics of Individuals with Type 1 Diabetes and Controls

	Control	T1DM	p values
Gender (m/f)	3/15	9/10	
Age (years)	30 ± 7	34 ± 8	0.09
Height (m)	1.70 ± 0.03	1.73 ± 0.02	0.4
BMI (kg/m^2^)	21 ± 2	28 ± 3	**<0.0001**
Normal weight/ over weight	18/0	4/15	
Waist circumference (cm)	75 ± 8	94 ± 15	**<0.0001**
Hip circumference (cm)	95 ± 6	108 ± 8	**<0.0001**
WHR	0.78 ± 0.1	0.87 ± 0.1	**0.03**
SBP (mmHg)	114 ± 15	128 ± 8	**0.001**
DBP (mmHg)	72 ± 9	80 ± 7	**0.003**
**Smoking:**			
Active smoker	0	3	
Past smoker	2	9	
Never smoked	16	7	
**Alcohol consumption**			
High risk	–	–	
Low risk	16	19	
No alcohol	2	–	
Diabetes duration (years)	–	18 ± 2	
**HbA1c (%)**			**<0.0001**
Normal (non-diabetic): <5.7%	5.1 ± 0.2	–	
Prediabetes: 5.7% to 6.4%	^−^	–	
Diabetes: 6.5% or higher	^−^	7.7 ± 0.9	
Total cholesterol (mmol/L) (Desirable: <5.0 mmol/L)	4.3 ± 0.6	5.3 ± 0.8	**0.0005**
HDL (mmol/L) Normal range: Men: >1.0 mmol/L Women: >1.2 mmol/L	1.55 ± 0.26	1.57 ± 0.33	0.9
LDL (mmol/L)	2.3 ± 0.6	3.2 ± 0.9	**0.002**
Triglycerides (mmol/L) (normal: <1.7 mmol/L)	0.9± 0.5	1.3 ± 0.6	0.09
ALT (U/L) Normal range: Men: ~10 to 40 U/L Women: ~7 to 35 U/L	18 ± 3	22 ± 8	0.05
AST (U/L) Normal range: Men: ~10 to 40 U/L Women: ~9 to 32 U/L	23 ± 7	21 ± 4	0.3
CRP (mg/L) Normal: <5 mg/L	0.5 ± 0	2.2 ± 2.3	**0.04**

**Notes**: Values are means ± SD, p <0.05 is significant. n=19 for T1DM and n=18 for Control.

**Abbreviations**: T1DM, type 1 diabetes mellitus; M/F, male/female; BMI, Body mass index; WHR, waist-to-hip ratio; SBP, systolic blood pressure; DPB, diastolic blood pressure; HbA1c%, hemoglobin A1C; HDL, high-density lipoprotein; LDL, low-density lipoprotein; ALT, alanine aminotransferase; AST, aspartate aminotransferase; CRP, C-reactive protein.

### Intestinal Permeability Markers

**Figure 1:**
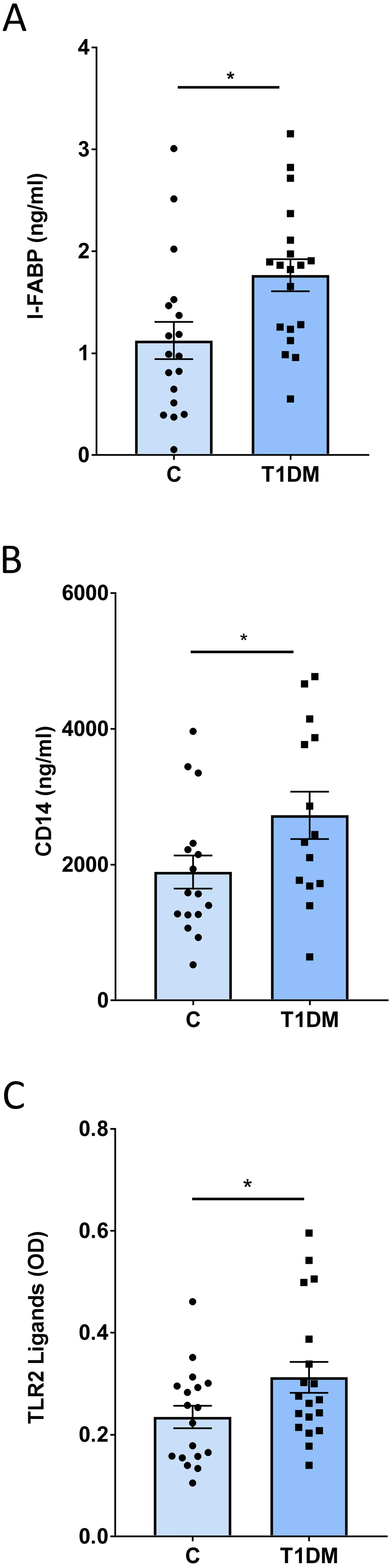
Markers of intestinal permeability and inflammation in the serum of control (**C**) and individuals with type 1 diabetes mellitus (T1DM). (**A**) I-FABP protein, (**B**) sCD14 protein, and (**C**) TLR2 ligand levels. *n= 18 controls and n=19* T1DM, except for sCd14 *n= 16 c* and n=14 T1DM. Values are shown as means ± SEM, *p< 0.05.

**Abbreviations**: I-FABP, intestinal fatty acid binding protein; sCD14, soluble cluster of differentiation 14; TLR, Toll-like receptor.

Concentrations of I-FABP in serum, being a marker of intestinal barrier function,^14^ were significantly higher in individuals with T1DM than in controls (95% CI: 0.15 to 1.13 ng/mL, mean difference: 0.64 ± 0.24, *p< 0.05, Figure 1A). Concentration of sCD14 (95% CI: 18.74 to 1690.00 ng/mL, mean difference: 835.60 ± 417.10, *p< 0.05) shown to be necessary for the recognition of ligands by TLR2,^15^ and concentration of TLR2 ligands (95% CI: 0.0012 to 0.1546 OD, mean difference: 0.0779 ± 0.0378, *p< 0.05) were also significantly higher in serum of T1DM participants (Figure 1B and C).

### Correlation Analysis and Multivariable Linear Regression Model

To test whether markers of intestinal barrier and TLR2 ligands as well as sCD14 levels are related to anthropometric or clinical parameters, correlation analyses within-groups were performed. No correlations were found except for I-FABP and WHR in the control group (*r* = 0.52 95% CI (0.05 to 0.80) p = 0.03 (Table 2)). As intestinal barrier function and TLR2 ligand concentration have been shown to be affected by age, BMI and sex,^16–18^ we next performed a multivariable linear regression analysis. While differences found for TLR2 ligand levels (control vs T1DM β = −0.152, 95% CI −0.296 to −0.007, p = 0.040) were still prevalent after adjustment for age, BMI, sex and smoking status, those for sCD14 (control vs T1DM β = −433.433, 95% CI −1990.79 to 1123.93, ng/ml, p = 0.570) and I-FABP (control vs T1DM β = −0.608, 95% CI −1.566 to 0.349 ng/mL, p = 0.204) were no longer found (Table 3).

**Table 2:** Within-Group Correlation of HbA1c%, BMI, Waist Circumference, and Waist-to-Hip Ratio with Intestinal Permeability Markers in Individuals with Type 1 Diabetes and Controls

Variable	Controls n	r (95% CI)	p	T1DM n	r (95% CI)	p
**TLR2**						
HbA1c (%)	17	−0.25 (−0.66 to 0.59)	0.17	19	0.22 (−0.26 to 0.61)	0.19
BMI (kg/m^2^)	18	0.11 (−0.39 to 0.56)	0.34	19	−0.18 (−0.60 to 0.31)	0.23
WC (cm)	18	0.05 (−0.43 to 0.50)	0.43	19	−0.09 (−0.53 to 0.39)	0.36
WHR	18	−0.03 (−0.49 to 0.45)	0.46	19	−0.12 (−0.56 to 0.37)	0.31
**I-FABP**						
HbA1c (%)	17	0.26 (−0.27 to 0.67)	0.16	19	−0.18 (−0.59 to 0.30)	0.23
BMI (kg/m^2^)	18	0.05 (−0.44 to 0.52)	0.85	19	0.17 (−0.33 to 0.59)	0.25
WC (cm)	18	0.29 (−0.21 to 0.68)	0.23	19	0.21 (−0.28 to 0.62)	0.19
WHR	18	0.52 (0.05 to 0.80)	0.03*	19	−0.07 (−0.52 to 0.41)	0.38
**sCD14**						
HbA1c (%)	15	−0.07 (−0.57 to0.47)	0.81	14	0.00 (−0.54 to0.55)	0.99
BMI (kg/m^2^)	16	−0.09 (−0.57 to 0.43)	0.73	14	−0.33 (−0.57 to 0.52)	0.46
WC (cm)	16	0.29 (−0.24 to 0.68)	0.28	14	−0.13 (−0.63 to 0.44)	0.33
WHR	16	0.34 (−0.20 to 0.72)	0.20	14	−0.13 (−0.63 to 0.44)	0.32

**Notes**: *p <0.05. n=15-18 for controls and n= 14–19 for T1DM.

**Abbreviations**: TLR, Toll-like receptor; I-FABP, intestinal fatty acid‑binding protein; sCD14, soluble cluster of differentiation 14; HbA1c%, haemoglobin A1C; BMI, Body mass index; WC, waist circumference; WHR, waist-to-hip ratio.

**Table 3:** Multivariable Linear Regression Analyses of TLR2, I-FABP, and sCD14 Levels Adjusted for Age, BMI, Sex and Smoking Status

Outcome	β (Control vs T1DM)	95%-CI	p-Wert
TLR2 (OD)	−0.152	−0.296 to −0.007	0.040*
I-FABP (ng/ml)	−0.608	−1566 to 0.349	0.204
sCD14 (ng/ml)	−433.43	−1990.79 to 1123.93	0.570

**Notes**: *p <0.05. n=15-18 for controls and n= 14–19 for T1DM.

**Abbreviations**: TLR, Toll-like receptor; I-FABP, intestinal fatty acid‑binding protein; sCD14, soluble cluster of differentiation 14.

## Discussion

Despite advances in its therapy, T1DM still frequently leads to serious long-term complications, including cardiovascular diseases, neuropathy, retinopathy, and nephropathy.^19^ The underlying mechanisms driving these effects remain unclear. Alterations of intestinal barrier function and a subsequent increase in Gram-negative bacteria‑derived endotoxin levels have been discussed to be among the causes of the sub-clinical, chronic low-grade inflammation frequently found in individuals with T1DM.^20^ It is not yet understood whether compounds derived from Gram-positive bacteria are also critical in the inflammatory processes found in individuals with T1DM. Here, by assessing markers of intestinal barrier function as well as sCD14 and TLR2 ligands, we found that in individuals with T1DM not only markers of intestinal barrier function such as I-FABP protein in serum but also sCD14 and TLR2 ligands are markedly higher than in healthy age-matched controls. These results are consistent with previous studies showing increased circulating I-FABP and sCD14 in T1DM;^21^,^22^ however, after adjustment for age, BMI, sex, and smoking status, no significant differences were observed for i-FABP or sCD14. T1DM was associated with significantly higher TLR2 levels. Indeed, overweight and obesity have been associated with impaired intestinal barrier integrity and increased intestinal permeability.^23^ While the underlying mechanisms have not yet been fully understood, a disruption of tight junction proteins has been related to changes in intestinal microbiota composition but also to nutritional factors.^17^,^24^,^25^ The mechanisms by which TLR2 ligands in individuals with T1DM are increased in the present study remain to be determined.

Also, in line with the findings of the present study, results of studies employing mice with streptozotocin‑induced diabetes show increased TLR2 expression and elevated levels of its endogenous ligands.^26^ Human studies report higher TLR2 protein levels in PBMCs in individuals with T1DM than controls.^27^ Furthermore, it has been shown that in TLR2-knockout mice the development of autoimmune diabetes is inhibited and insulitis is lower compared with wild-type animals.^28^ Besides its function in glycaemic control, insulin has been shown to support intestinal barrier integrity^29^ and suppresses innate immune activation. Also, even under insulin therapy, T1DM has been shown to be related to a persistent TLR2/TLR4 activation,^4^ possibly driven by poor glycaemic control and advanced glycation end products. In the present study, the mean duration of T1DM was 18 years. Intestinal barrier dysfunction has been reported in both newly diagnosed and long-standing T1DM, suggesting that while it is an early feature of the disease it also persists over time.^30^ Also, chronic hyperglycaemia has been shown to contribute to TLR activation and low-grade inflammation by inducing TLR2 and TLR4 expression and downstream inflammatory signalling.^31^

Interestingly, in the present study employing a correlation analysis within-group, no significant correlations were found between markers of intestinal barrier function and TLR2 ligands with HbA1C%, BMI, WC and WHR. The data, which are in line with findings in patients with type 2 diabetes^32^ support the hypothesis that intestinal barrier dysfunction may be related to glycemic control and adiposity.^17^ Indeed, it has repeatedly been shown that metabolic diseases like type 2 diabetes and MASLD are related to changes of intestinal barrier function and higher bacterial endotoxin as well as TLR2 ligand concentration.^33^ The lack of correlation of sCD14 with clinical markers might have resulted from its regulation which is solely based on gene transcription but also shedding from cells.^34^,^35^ However, molecular mechanisms underlying the association found in the present study as well as the lack of association for sCD14 remain to be determined. Taken together, these data lend further support to the hypothesis that intestinal barrier function is impaired in patients with T1DM and that it may not only result in an increased translocation of bacterial endotoxin derived from Gram-negative bacteria but also of Gram-positive bacteria.

When interpreting the results of the present study, several limitations have to be considered. One limitation is that the control group was not matched for BMI and data were obtained from participants recruited at a single center only. Also, only one individual with T1DM used an insulin pump and, in most individuals, HbA1c levels were elevated. It remains to be determined whether, in individuals with type 1 diabetes mellitus who have a normal HbA1c, markers of intestinal barrier function and TLR2 ligand levels are at the level of healthy controls. Additionally, intestinal permeability was only assessed indirectly. Alternative methods such as a polyethylene glycol-based test could offer greater insight. Furthermore, the TLR2 assay employed in the present study only detects total TLR2 ligands. Lastly, the small sample size warrants caution when interpreting the findings as the study was sufficiently powered only to detect large between-group differences and its cross-sectional design does not allow conclusions regarding causality. Also, when performing a multivariable linear regression analysis effect of sex and BMI being significantly different between groups cannot be ruled out.

## Conclusion

In summary, while being a small study with control subjects only matched for age but not for BMI, our data suggests that in overweight T1DM individuals—who have poorly controlled T1DM—TLR2 ligands are elevated and markers of intestinal barrier function are altered. Larger studies are needed to confirm these findings and to determine underlying mechanisms.

## Data Availability

The data presented in this study are available upon reasonable request from the corresponding author due to legal and ethical reasons.
